# A new approach to physical activity maintenance: Rationale, design, and baseline data from the Keep Active Minnesota trial

**DOI:** 10.1186/1471-2318-8-17

**Published:** 2008-07-25

**Authors:** Nancy E Sherwood, Brian C Martinson, A Lauren Crain, Marcia G Hayes, Nicolaas P Pronk, Patrick J O'Connor

**Affiliations:** 1 HealthPartners Research Foundation, Minneapolis, MN, USA; 2 Division of Epidemiology and Community Health, School of Public Health, University of Minnesota, Minneapolis, MN, USA; 3 HealthPartners Health Behavior Group, Minneapolis, MN, USA

## Abstract

**Background:**

Since many individuals who initiate physical activity programs are highly likely to return to a sedentary lifestyle, innovative strategies to efforts to increase the number of physically active older adults who successfully *maintain *beneficial levels of PA for a substantial length of time are needed.

**Methods/Design:**

The Keep Active Minnesota Trial is a randomized controlled trial of an interactive phone- and mail-based intervention to help 50–70 year old adults who have recently increased their physical activity level, maintain that activity level over a 24-month period in comparison to usual care. Baseline, 6, 12, and 24 month measurement occurred via phone surveys with kilocalories expended per week in total and moderate-to-vigorous physical activity (CHAMPS Questionnaire) as the primary outcome measures. Secondary outcomes include hypothesized mediators of physical activity change (e.g., physical activity enjoyment, self-efficacy, physical activity self-concept), body mass index, and depression. Seven day accelerometry data were collected on a sub-sample of participants at baseline and 24-month follow-up.

**Discussion:**

The Keep Active Minnesota study offers an innovative approach to the perennial problem of physical activity relapse; by focusing explicitly on physical activity maintenance, the intervention holds considerable promise for modifying the typical relapse curve. Moreover, if shown to be efficacious, the use of phone- and mail-based intervention delivery offers potential for widespread dissemination.

**Trial registration:**

ClinicalTrials.gov Identifier: NCT00283452.

## 1. Background

Despite substantial data documenting the health benefits of physical activity [1-18], sedentary behavior remains a significant public health problem that is particularly prevalent among older adults. The 2005 Behavioral Risk Factor Surveillance System (BRFSS) documents that among adults age 45–54, more than half (52%) were obtaining less than recommended levels of physical activity, with the same being true for 55% of adults age 55–64 [19]. With a few exceptions [20,21], most intervention efforts have remained focused on PA initiation [22-32]. However, since ongoing participation in PA is necessary to sustain health benefits, complementary strategies to increase the number of sedentary individuals who *initiate *PA and efforts to increase the number of physically active individuals who successfully *maintain *beneficial levels of PA for a substantial length of time are needed. The importance of focusing attentions specifically on maintaining PA, is underscored by reports that roughly half of older adults who initiate a program of PA discontinue within three months [11]. These data, coupled with the observation that prevalence of sedentary behavior increases with age [19] suggests that population levels of PA may be substantially increased by preventing currently active individuals from falling below recommended levels of physical activity. More programs are needed for older adults that focus on maintenance, incorporate moderate intensity PA and are simple, convenient to engage in, relatively inexpensive, and noncompetitive [13].

Lack of data on the efficacy of low-cost interventions that have high penetration into target populations remains a hurdle to widespread dissemination of population-based interventions to increase levels of PA. The Keep Active Minnesota project addresses this need. Several aspects of our intervention should increase its efficiency and credibility, relative to other interventions, making it uniquely suited for widespread dissemination. First, by employing a phone- and mail-based strategy of intervention outside of the clinical setting, we do not add a burden to primary care providers. Second, by focusing on the older adult population, we target those at ages where maintenance of physical activity may yield particularly large benefits in terms of disease prevention and health improvement. Third, by supporting PA maintenance among recently active older adults, we are targeting individuals during a life-course stage where they are at increased risk for becoming sedentary. Fourth, those who have self-initiated a recent increase in physical activity have signaled their willingness and interest in maintaining healthy behaviors, which may make them more adherent to behavioral recommendations. Finally, because maintenance efforts may require fewer resources than interventions promoting initiation, the overall resource demands and per-unit costs of delivering our program should be lower than comparable initiation programs.

We address this research gap by evaluating the efficacy of a population-based approach to promoting PA *maintenance *among 50–70 year old adults who recently increased their physical activity level. The primary goal of the Keep Active Minnesota study is to assess the extent to which an interactive phone- and mail-based intervention helps participants maintain the level of activity they reported at baseline, over a period of two years in comparison to usual care. This paper describes the design and baseline data of the ongoing Keep Active Minnesota physical activity maintenance trial.

## 2. Methods

### 2.1 General design

**Figure 1:**
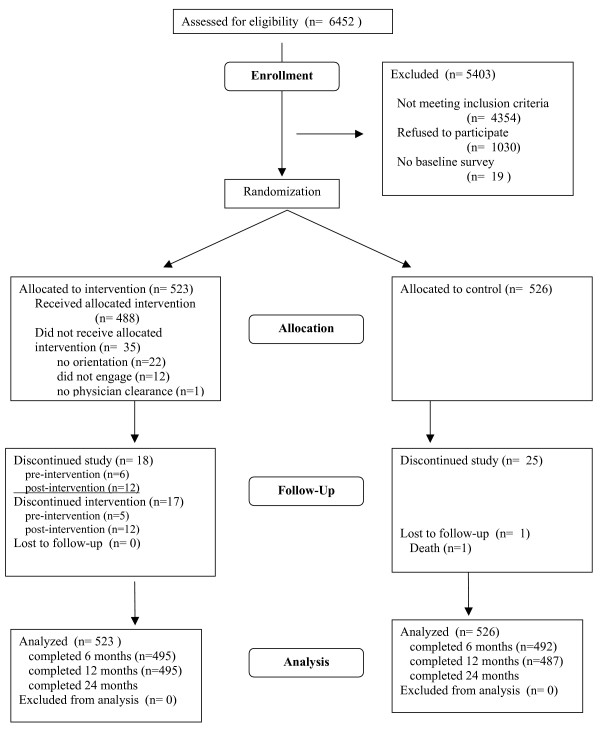
CONSORT diagram to describing flow of participants through the trial.

The goal of Keep Active Minnesota was to recruit members of a large managed care organization (HealthPartners, hereafter HP) who had recently increased their physical activity level outside of a formal research study, and randomize them to one of two study groups: a telephone- and mail-based intervention designed to promote physical activity maintenance and a usual care control group who will not receive any physical activity intervention or advice beyond what is typically provided to health plan members. Absolute level of physical activity and maintenance of physical activity, defined as physical activity level relative to baseline, are the primary study outcomes. Figure 1 depicts the CONSORT diagram for the trial.

### 2.2 Recruitment and screening

The goal of recruitment was to obtain baseline data from 1,000 male and female HP members ages 50–70 who were eligible and interested in participating in the intervention study. A two-phase recruitment process was used. In the first phase, a sampling frame of 104,000 HP members was identified using administrative data and the eligibility criteria described below in the eligibility section. In mid July 2004, a screening survey was administered by mail to a random sample of 4,000 age-eligible individuals, to provide data to characterize the population from which the intervention sample was being recruited. These individuals received a letter describing the study and a six page survey which we use elsewhere (manuscript in preparation) to assess similarities and differences between study enrollees and the sampling frame from which they were recruited. This survey also included a brief set of eligibility screening questions. Participants who completed the survey and met eligibility based on PA criteria were called, received information on the second phase of the study and were asked if they were interested in participating.

In the second phase, recruitment proceeded both via direct mail recruitment starting in December 2004, and via self-referral, starting in March 2005. For the direct mail recruitment efforts, a cover letter similar to that used in the first recruitment phase was sent; no survey was included, however, the back of the cover letters contained several brief screening questions to determine eligibility and interest. Recipients were asked to answer these questions and return them. Upon receipt of these screeners, eligible participants who indicated interest in the study were contacted by phone. Two strategies were implemented to increase representation of racial/ethnic minorities in the study population. First, direct mailings of invitation letters and eligibility screeners were sent to all age-eligible health plan members who were identified as racial/ethnic minorities in the HP administrative database. Because collection of information on race/ethnicity had only recently started in the health plan, the number of potentially eligible individuals so identified was only about 2,100 out of an age eligible pool of roughly 104,000. Second, geographically targeted direct mailings were conducted to more than 2,000 age-eligible health plan members who reside in census tracts in which minority individuals are over-represented (>= 40% racial/ethnic minorities, based on Census 2000 counts). These strategies were completed in the first four direct mail waves, ending in March of 2005.

The final two waves of direct mailings were targeted to eligible health plan members who had engaged in one of two health plan programs that promote physical activity. In May, mailings were sent to participants in a program that pays a rebate to health plan members who visit participating health clubs a minimum of eight times per month. In July, mailings were sent to health plan members who participate in a mail and web based physical activity program that uses a pedometer.

As a third method of recruitment, inexpensive forms of advertising were used to generate "self-referrals" to intervention staff who conducted phone-based, real-time eligibility screening of interested individuals. Brief descriptions of the study and eligibility criteria were placed in a variety of print, email and web media. Venues included a health newsletter that is sent to all HP members; the HP and HealthPartners Research Foundation (HPRF) web sites; an electronic newsletter to HP employees; posters and brochures at all metropolitan YWCA's and YMCA's; a targeted email from a large employer to all age eligible employees with HP insurance; and two other large employers included the descriptions of the study in electronic newsletters to employees.

To be eligible, participants had to be between the ages of 50–70 years old, enrolled in the health plan for at least 11 of the 12 months prior to screening for study eligibility, and have increased their physical activity level either on their own or with the support of a program during the past year to a minimum of 30 minutes of moderate or vigorous PA a day at least 2 days per week on average over the past four weeks. The minimum of two days per week of moderate intensity PA is a clinically relevant cutoff, since regular PA at this level has been shown to generate significant improvements in functional capacity, fasting insulin levels, and other health-related variables [33-35] and to reduce risk of Type 2 diabetes in women [36]. Participants were excluded if they had a modified Charlson comorbidity score > 3 (a standard index of comorbidity calculated using prior year diagnoses of a broad range of serious medical conditions) [37,38], or had diagnoses of coronary heart disease (CHD), congestive heart failure (CHF), atrial or ventricular arrhythmias, cardiac arrest, or had an implantable defribillator.

Regardless of recruitment method, all potential participants were contacted by telephone to confirm their interest in study participation and to conduct an initial consent discussion. Following this discussion, consent forms were mailed to interested individuals who were asked to read, sign, and return a consent form. The consent form was reviewed and approved by the Regions Hospital Human Subjects Review Board. When completed consent forms were received a baseline telephone interview (see Section 2.7 below) was scheduled with the participant.

### 2.3 Randomization

Upon completion of the baseline telephone interview, participants were randomized by the study coordinator as they enrolled according to a schedule pre-determined by the study statistician and unobservable to the staff conducting randomization, based on a random number table embedded in the backend of the recruitment tracking database. Blocks of 20 were used to maintain study arm balance throughout the recruitment period. 1,049 subjects were randomized either the PA treatment condition (KAM) or a usual care control group (UC).

### 2.4 Intervention background

PA is a complex behavior with multiple determinants and pathways to change [39,40]. Historically, behavior change theory, research and intervention have primarily focused on initiation as opposed to maintenance. However, the importance of behavior change maintenance and the recognition that mechanisms underlying maintenance likely differ from those underlying initiation have been receiving increased attention in recent years. Three perspectives relevant to PA maintenance that inform our intervention include the Transtheoretical Model (TTM) [41-43], Rothman's theory regarding differential decision criteria for initiation versus maintenance [44], and the relapse prevention model [45]. We also look to the extensive literature on behavioral determinants of PA and activity maintenance to develop our intervention focus. Finally, since no single theory encompasses all factors related to PA maintenance [46] we use social cognitive theory (SCT) [47] as an organizing framework. Given its multidimensional emphasis on personal, behavioral and environmental factors, SCT provides a useful framework for accommodating the complexity of factors thought to influence PA maintenance.

**Figure 2:**
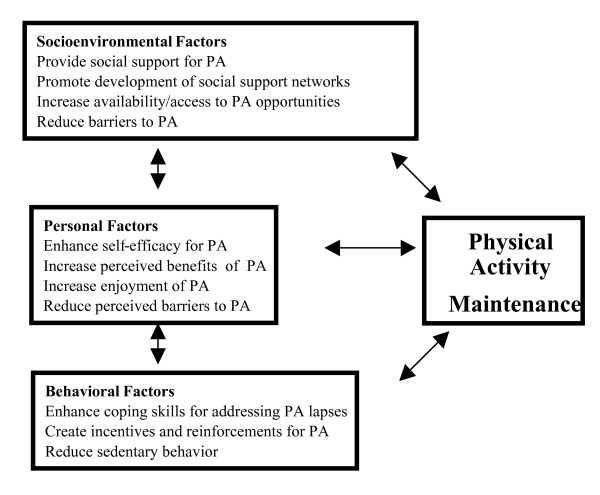
SCT-based Conceptual Model for PA Maintenance.

Key constructs relevant to PA maintenance, depicted in Figure 2, include self-efficacy, perceived benefits of PA, coping skills for dealing with lapses, social support, and access-availability of PA opportunities. These measures provide an opportunity to gain insight into mediating factors associated with PA maintenance, an important, but understudied component of behavior change research [48,49].

### 2.5 KAM intervention description

Participants randomized into the intervention were offered an interactive telephone and mail-based physical activity support program. After randomization, participants assigned to the intervention arm were invited to attend an in-person orientation to the study. Sessions started in October of 2004 and ended in November of 2005. There were a total of 13 sessions and attendance ranged from approximately 10 to 50 participants per session. During the orientation participants were introduced to the study staff, received information on the study's goals and procedures, picked up their study materials and, when possible, made an appointment for their first session with the phone coach. Approximately two-thirds of intervention study participants attended the in-person orientation. Those unable to attend in person were received their study materials via mail and took part in a phone based orientation with one of the activity coaches.

The core component of the intervention was a seven session course delivered over the phone by activity coaches with a background in exercise science and training in behavior change theory. Each course workbook session included topics to be covered during the phone coaching session, instructional material, assignments for participants to work through on their own and goal setting. Participants were encouraged to use their pedometer to monitor their physical activity, however, they could also choose to self-monitor their physical activity in whatever way would be useful to them (e.g., minutes, miles). Course session topics included: the benefits of physical activity; goal setting and the development of an action plan; a discussion of types of physical activity and exercise; overcoming barriers to physical activity, problem solving and enhancing self-efficacy; social support; healthy eating; relapse prevention; and developing an action plan for long term maintenance.

The sessions were scheduled at the participant's convenience, designed to last about 20 minutes, to take place about twice a month and to be tailored for individual participants.

The course sessions were set up so that goals from the last session were initially reviewed along with any questions or concerns about previous topics. Then the topic of the session along with the accompanying homework was discussed. Activity coaches provided feedback and encouragement, helped with problem solving and encouraged the participant to revise their physical activity goals, if needed.

Following completion of the course, participants receive monthly follow-up calls for the remainder of the first year of study participation and then bi-monthly calls for the second year. Additional intervention components include motivational challenges, group sessions, and a lending library of physical activity resources.

Intervention participants were invited to engage in three motivational challenges during the course of their time in KAM. The challenges were designed to support the course objectives and timed to occur at six month intervals, with the first challenge starting soon after the subject finished the bi-weekly phone course. At the start of the challenge, participants were sent a flyer describing the challenge and a tracking form to document contest participation that could be mailed in a provided envelope before the contest deadline. Participants were also encouraged to share a story about lifestyle behavior changes they had made related to the challenge. The challenge topics were cross-training, tracking healthy eating and stress reduction strategies and taking a virtual walk to a popular and well-known Minnesota destination, via pedometer or minutes walked; the numbers of participants who engaged in each challenge were 91, 118 and 156, respectively.

Small prizes were offered for individual participation, for the "wave" (defined by the month the participant completed their in-person or phone orientation) with the most participants and for the individual submitting the most engaging story. At the end of each challenge time period, a newsletter was sent that announced the winners in each category. The newsletter also contained the winning story, a profile of a KAM participant as well as a profile of a KAM study team member and a list of physical activity opportunities in the Twin Cities metro area for the next six months.

If subjects wanted to try a new type of exercise or were in need of motivation, they were offered resources from a physical activity toolbox, a lending library of books, videos and DVD's. During the course of the study more than 70 items were mailed to subjects.

Four different group sessions featuring outside guest-speakers were offered during the second year of the study. A majority of study participants were still active in the study at that time. The sessions were designed to offer advice and support in a different venue than the course. Session topics included sports medicine, healthy eating, staying active in the winter and bicycling. Attendance varied with the topic and season, but approximately 50 subjects, on average, attended each group session.

### 2.6 Usual care description

Participants randomized into the Usual Care (UC) condition received information about the 10,000 steps physical activity program offered by the healthplan after their baseline phone survey and 4 newsletters focused on general health and wellness during their two years of study participation.

### 2.7 Measurement

All primary and secondary outcome measures were collected during a 45 minute telephone interview scheduled at a participant's convenience and administered prior to randomization, and 6, 12, and 24 months later. Participant responses were recorded by the interviewer onto an optically scan-able form that was scanned following administration, so that data were immediately available in the main study database.

### 2.8 Primary and secondary physical activity outcomes

The primary outcome measures for this study are kilocalories expended per week on a range of activities (total kcal/wk) and kilocalories expended per week on a subset of moderate and vigorous activities (moderate kcal/wk). Both kcal expenditure measures are computed using the CHAMPS instrument, designed for use in adult populations such as this one to assess the self-reported frequency and duration of a range of common activities and convert these reports into weekly kcal expenditure [50]. The CHAMPS instrument has demonstrated acceptable reliability with ICCs for moderate intensity activities of 0.67, 0.76, and 0.81–0.88 at six months, two weeks, and one week, respectively. Higher intensity activities demonstrate more modest ICCs of 0.66, 0.62, and 0.34–0.45 at six months, two weeks, and one week, respectively [51,52]. The instrument has also demonstrated adequate discriminate and construct validity, correlates well with other measures of physical activity and is sensitive to change [52].

Sample size was based on that which would be needed to detect a time (24 month vs. baseline observation) by treatment (KAM vs. UC) interaction at .80 power (two-tailed, alpha = 0.05) on the total kcal/wk variable in a two group repeated measures ANOVA. We assumed a common standard deviation of 1500 kcals/wk at each of 4 time points and a first order autoregressive residual covariance structure. These parameters suggested that N = 349 per study arm would be needed to detect the interaction of primary interest. Assuming non-differential 70% retention across study groups, we recruited N = 500 per arm.

Additional PA outcomes assessed include whether participants maintained PA at the follow-up measurement points, defined as moderate kcal expenditure at least 1500 kcal/wk and at least 80% of that expended at baseline and whether participants met CDC/ACSM physical activity recommendations of 30 minutes of moderate activity 5 or more days per week (moderate); 20 minutes of vigorous activity at least 3 days per week (vigorous); and moderate or vigorous activity recommendations [53]. A randomly selected sub-sample of enrolled study participants (50 from each treatment group) had their PA monitored via the MTI Actigraph (Manufacturing Technologies, Inc. Fort Walton Beach, previously referred to as the Computer Science and Applications (CSA) Monitor) at baseline and 24 month follow-up. Body mass index (BMI; kg/m^2^) was calculated from baseline and 6 month self-reported weight and baseline height.

### 2.9 Demographic characteristics

At baseline, participants provided information about their age, sex, race/ethnicity, educational attainment, household income, employment status and marital status.

### 2.10 Physical activity mediators and moderators

Hypothesized mediators and moderators of physical activity measured included: 1) a 4-item measure of Physical Activity Enjoyment adapted from a previous measure by Motl et al [23], 2) a 5-item measure of *Physical Activity Self-Concept *adapted from the Athletic Identity Measurement Scale [54], 3) the The 10-item Social Support for Exercise Behavior Questionnaire [55], 4) a 12-item version of the *Barriers Self-Efficacy Scale *[56], 5) a 13-item scale measuring the perceived benefits of physical activity adapted from [57], and 6) the 11-item short-form of the Center for Epidemiological Studies Depression symptoms scale [58].

### 2.11 Analyses

Mixed model regression (time within participant, unstructured covariance structure, restricted maximum likelihood estimation) will be used to test the hypothesis that KAM participants maintained kcal expenditure from baseline to 6,12, and 24 months, relative to a drop in kcal expenditure among the UC participants. Total and moderate kcal will be separately predicted from the time at which kcal was measured, which varies within participants, and randomized treatment group (KAM, UC), which varies across participants. This intent to treat approach will ensure that all available kcal observations, excluding those greater than 5 SD above median, from all randomized participants will be used to estimate model parameters.

For this report, 2-sided t-test, and chi-square statistics were used to compare the baseline characteristics of: 1) participants recruited for the study through direct mail versus self-referral; and 2) participants randomized to the intervention and control groups.

## 3. Results

Table 1 shows participant characteristics and physical activity level according to recruitment source; direct mail and self-referral. Participants who responded to a direct mailing about the study were less likely to be female and more likely to report "fair" or "poor" health status compared to those who self-referred themselves into the study after learning about the study through print, email or web media advertisement. No other statistically significant demographic or baseline physical activity characteristics differentiated direct mail and self-referral participants.

**Table 1:** Participant Characteristics and PA as Mean and Standard Error or Percent by Source of Recruitment

	Direct Mail	Self-Refer	All
N	824	225	1049

age at baseline	57.1(.18)	57.2(.34)	57.1(.16)

female	69.5	82.7***	72.4

BMI, kg/m^2^	27.6(.19)	27.5(.38)	27.6(.17)

White	94.2	97.3	94.9

Hispanic/Latino	1.9	1.8	1.8

employed full time	76.2	79.1	76.8

4 year degree or more	66.3	68.4	66.7
			
functional health status fair or poor	7.0	3.1*	6.2

kcal/wk,	4721	4679	4712
total	(91)	(155)	(79)

kcal/wk,	2837	2731	2815
moderate or vigorous	(72)	(125)	(63)

moderate activity	23.5	28.4	24.6
30 minutes, 5/week			

vigorous activity	35.7	36.4	35.8
20 minutes, 3/week			

moderate or vigorous	48.8	55.1	50.1

* self-refer vs. mail, p < .05

*** self-refer vs. mail, p < .001

As documented in Table 2, at baseline the typical participant was about 57 years old, female, White, overweight (BMI ≥ 25 kg/m^2^), working full time, and college educated. Few participants reported fair or poor functional health status. Randomization successfully resulted in study groups that were similar on most demographic, physical activity, and psychosocial characteristics. The only observed demographic difference between the groups was that fewer KAM participants self-identified as White (95.6 vs. 92.4%, p < .05). Participants assigned to the KAM group were less likely to report engaging in moderate physical activity a minimum of five times per week, but reported higher levels of social support for physical activity from family members.

**Table 2:** Participant Characteristics and PA as Mean and Standard Error or Percent by KAM and Usual Care Groups

	UC	KAM	All
N	526	523	1049

age at baseline	57.1(.22)	57.1(.22)	57.1(.16)

female	71.8	72.9	72.4

BMI, kg/m^2^	27.7(.24)	27.5(.23)	27.6(.17)

White	96.2	93.5*	94.9

Hispanic/Latino	1.2	2.5	1.8

employed full time	76.6	77.1	76.8

4 year degree or more	65.8	67.7	66.7

functional health status fair or poor	5.7	6.7	6.2

kcal/wk,	4781	4643	4712
total	(114)	(109)	(79)

kcal/wk,	2898	2730	2815
moderate or vigorous	(94)	(83)	(63)

moderate activity	27.8	21.4*	24.6
30 minutes, 5/week			

vigorous activity	35.0	36.7	35.8
20 minutes, 3/week			

exercise efficacy	4.59	4.59	4.59
all items	(.02)	(.02)	(.01)

support: family	2.73	2.82*	2.78
all items	(.03)	(.03)	(.02)

support: friends	2.60	2.65	2.63
all items	(.03)	(.03)	(.02)

enjoyment	4.12	4.08	4.10
	(.03)	(.04)	(.03)

PA Self-concept	3.86	3.84	3.85
	(.04)	(.03)	(.02)

PA self-efficacy	55.9	56.0	56.0
(1–100 scale)	(.79)	(.76)	(.55)

depression	0.35	0.38	0.36
mean of items	(.01)	(.01)	(.01)

* KAM vs. UC, p < .05

*** KAM vs. UC, p < .001

## 4. Discussion

This paper describes the design of the Keep Active Minnesota (KAM) trial and the baseline characteristics of the KAM intervention and usual care groups. This randomized controlled trial will evaluate the efficacy of a relatively low intensity phone and mail-based intervention designed to promote *maintenance *of physical activity among 50–70 year old adults who have recently increased their physical activity level. It may seem counterintuitive to expend effort to help those who have already initiated greater physical activity on their own maintain those efforts. However, just as smoking cessation is frequently not accomplished with a first attempt, so too, attempts to sustain a healthy level of PA may require multiple attempts. Moreover, even highly active individuals experience lapses in the face of high risk situations [59] and those who have recently increased their physical activity level may be particularly vulnerable during the period of time that it takes for them to move from initiating a new behavior to it becoming a well-established behavior or habit. Providing a modest level of effort to assist such individuals may well yield benefits that are more than commensurate with the effort expended. For some, it may be additional information or help in problem solving that will help to take them through this period. For others, it may simply be having an external agent who they know is working on their behalf, and to whom they feel a sense of connection or accountability that may do the trick – sustaining them in their efforts while they develop the skills to problem solve and overcome the inevitable barriers to physical activity.

To enroll in the trial, participants had to have increased their physical activity level during the past year to a minimum of 30 minutes of moderate or vigorous PA a day at least 2 days per week. Maintenance of this relatively modest physical activity level has the potential to yield important health benefits. Including improvements in functional capacity, fasting insulin levels, and reduce risk of Type 2 diabetes [33-35]. Although all KAM participants were required to meet the two day per week threshold, there was considerable variability in the frequency and intensity of reported physical activity. Subgroup analyses will examine whether the KAM intervention is differentially effective based on baseline physical activity level and physical activity history; secondary analyses will also be informative with respect to the dose of physical activity necessary for weight maintenance in this population.

Since most randomized trials of physical activity interventions focus primarily on initiation, with attention paid to maintenance when the novelty of the intervention may have worn off and adherence may be declining, recruitment to a maintenance-focused intervention provides an opportunity to engage people during the time period when they may be otherwise vulnerable to lapses in physical activity. Both the content and delivery of the KAM intervention were tailored for addressing maintenance. Telephone-based counseling has an increasing evidence base [60] and may be particularly suitable for a maintenance-focused intervention given that it is a flexible and relatively lower intensity intervention. The KAM intervention is also based on a theoretical model specifically developed to address issues related to PA maintenance which integrates principles of Bandura's Social Cognitive Theory (SCT) [47] and relapse prevention theory [61]. Intervention strategies were weighted heavily toward self-management, including cognitive (goal setting, identification of barriers and problem solving), behavioral (self-monitoring through use of pedometers & log-books, use of environmental cues), and environmental (phone coach support, development and leverage of social support) strategies.

Strengths of the KAM trial are its unique focus on maintenance, implementation of theory-based intervention and the use of phone- and mail-based delivery mechanism which due to its relatively low cost may lead to a high potential for dissemination if the intervention is shown to be efficacious over the long term. Limitations of the KAM study include the ability to generalize to a broad population of adults given the focus on 50–70 year olds and limited racial/ethnic diversity of the study sample. Although the intervention appealed to a large number of those eligible to enroll in the study, multiple strategies are needed to assure the broad population penetration needed to increase overall population levels of PA. Despite these limitations, the KAM study offers an innovative approach to the perennial problem of physical activity relapse. By focusing explicitly on physical activity maintenance, the KAM intervention holds considerable promise for modifying the typical relapse curve.

## Competing interests

The authors declare that they have no competing interests.

## Authors' contributions

NES drafted the manuscript, directed the intervention, and assisted with conceptualization and implementation of all aspects of the study. BCM, as Principal Investigator, led the project team and the development and implementation of the study and assisted with the writing of the manuscript. ALC assisted with conceptualization and implementation of all aspects of the study and conducted the analyses for the manuscript. MGH assisted with implementation of all aspects of the study and assisted with the writing of the manuscript. NPP assisted with the conceptualization of the study. PJO'C assisted with the conceptualization and implementation of the study and edited the manuscript. All authors read and approved the final manuscript.

## Pre-publication history

The pre-publication history for this paper can be accessed here:


